# Impact of the California Lead Ammunition Ban on Reducing Lead Exposure in Golden Eagles and Turkey Vultures

**DOI:** 10.1371/journal.pone.0017656

**Published:** 2011-04-06

**Authors:** Terra R. Kelly, Peter H. Bloom, Steve G. Torres, Yvette Z. Hernandez, Robert H. Poppenga, Walter M. Boyce, Christine K. Johnson

**Affiliations:** 1 School of Veterinary Medicine, Wildlife Health Center, University of California Davis, Davis, California, United States of America; 2 Department of Fish and Wildlife, University of Idaho, Moscow, Idaho, United States of America; 3 Wildlife Investigations Laboratory, California Department of Fish and Game, Rancho Cordova, California, United States of America; 4 Molecular Biosciences, School of Veterinary Medicine, University of California Davis, Davis, California, United States of America; University of Lethbridge, Canada

## Abstract

Predatory and scavenging birds may be exposed to high levels of lead when they ingest shot or bullet fragments embedded in the tissues of animals injured or killed with lead ammunition. Lead poisoning was a contributing factor in the decline of the endangered California condor population in the 1980s, and remains one of the primary factors threatening species recovery. In response to this threat, a ban on the use of lead ammunition for most hunting activities in the range of the condor in California was implemented in 2008. Monitoring of lead exposure in predatory and scavenging birds is essential for assessing the effectiveness of the lead ammunition ban in reducing lead exposure in these species. In this study, we assessed the effectiveness of the regulation in decreasing blood lead concentration in two avian sentinels, golden eagles and turkey vultures, within the condor range in California. We compared blood lead concentration in golden eagles and turkey vultures prior to the lead ammunition ban and one year following implementation of the ban. Lead exposure in both golden eagles and turkey vultures declined significantly post-ban. Our findings provide evidence that hunter compliance with lead ammunition regulations was sufficient to reduce lead exposure in predatory and scavenging birds at our study sites.

## Introduction

Almost 20 years have passed since implementation of the nationwide ban of lead shot for waterfowl hunting in the United States [1]. Prior to this regulation, it was estimated that 2–3% of the mortality in the fall waterfowl population in North America could be attributed to lead poisoning [2], [3]. In Canada and the United States, an estimated 10–15% of documented post-fledging mortality in bald (*Haliaeetus leucocephalus*) and golden eagles (*Aquila chrysaetos*) was attributed to lead poisoning from ingestion of lead shotgun pellets in waterfowl wounded or killed by lead ammunition [4], [5]. In response to concerns regarding lead related mortality in waterfowl populations and secondary poisoning of the bald eagle, a federally mandated phase-in of non-lead shot was initiated in heavily impacted wetlands in North America in 1986, and, in 1991, a ban of lead-based ammunition for waterfowl hunting went into effect nationwide [1].

Since implementation of the ban, several studies have assessed its effectiveness in reducing lead exposure in impacted waterfowl populations. Six years following its initiation, Anderson et al., 2000 [6] estimated that the ban of lead-based ammunition reduced lead related mortality of mallards in the Mississippi Flyway by 64% and saved 1.4 million ducks nationwide in the fall migration of 1997. There was also a documented 44% decline in the prevalence of elevated blood lead exposure in American black ducks (*Anas rubripes*) in the Mississippi Flyway following the ban [7]. While this regulation significantly reduced lead pellet ingestion and estimated lead-associated mortality in North American waterfowl, it did not result in decreased numbers of lead-poisoned eagles presenting from multiple states to a raptor rehabilitation center in Minnesota during a five year period following the ban [8]. The authors attributed these ongoing lead poisoning cases in part to ingestion of fragmented lead bullets in discarded viscera from field processed deer, as the highest rates of eagle poisoning coincided with the deer hunting season.

Scavenging and predatory birds are highly susceptible to lead intoxication when they consume embedded lead shot or fragmented lead bullets in un-retrieved hunter-killed carcasses, discarded viscera, or hunter-crippled animals, as has been observed with bald eagles preying upon shot and injured waterfowl [9]–[13]. Upon impact, lead-based projectiles can produce hundreds of small fragments resulting in contaminated animal carcasses and gut-piles that serve as carrion for scavengers [14]–[17].

Lead poisoning played a role in the decline of the endangered California condor (*Gymnogyps californianus*) population in the 1980s [18] and still remains a major barrier to population recovery [19]. Consequently, lead ammunition used for most hunting activities in the range of the condor in California was banned as of July 2008; the first policy of its kind to ban lead ammunition for the take of big game in North America. Stakeholder groups continue to be highly polarized on this issue, with some arguing that there is a lack of scientific evidence to warrant regulation of lead-based ammunition for hunting [20].

Monitoring of lead exposure in condors and other scavenging and predatory wildlife species is essential for evaluating trends and determining whether existing restrictions of lead-based ammunition will be effective in reducing lead exposure. Like California condors, scavenging birds, such golden eagles and turkey vultures, are indicator species that can be used long-term to monitor the effectiveness of the lead ammunition ban. Golden eagles are abundant in the southern aspect of the condor range in California and serve as a sentinel species for lead exposure in this area. Despite their predatory nature, golden eagles will scavenge carrion readily especially during winter months [21], and may also target hunter-crippled small mammal prey. Because golden eagles utilize both live prey and carrion as food sources, they may be less sensitive as an indicator of lead exposure from spent ammunition compared to other scavenging species, except during the winter months when they are more dependent on carrion. Turkey vultures (*Cathartes aura*) are scavengers and feed on a wide array of carrion [22]. In another study, we found that blood lead concentrations in turkey vultures were significantly associated with big game hunting activities in California and were elevated in the central portion of the condor range, where there is high wild pig hunting intensity [23]. Turkey vultures are abundant in this area and serve as a good indicator species for lead exposure. The objective of this study was to evaluate the effectiveness of the California lead ammunition ban in decreasing blood lead exposure in scavenging birds by comparing golden eagle and turkey vulture blood lead concentrations before and after implementation of the ban.

## Materials and Methods

### Ethics statement

Animal capture and sampling protocols were covered under federal and state permits (U.S. Geological Survey (USGS) federal bird banding permit # 20431 and California Department of Fish and Game (CDFG) scientific collecting permit # 000221) and approved by the University of California, Davis Institutional Animal Care and Use Committee (protocol # 07-12955).

### Study site selection

**Figure 1:**
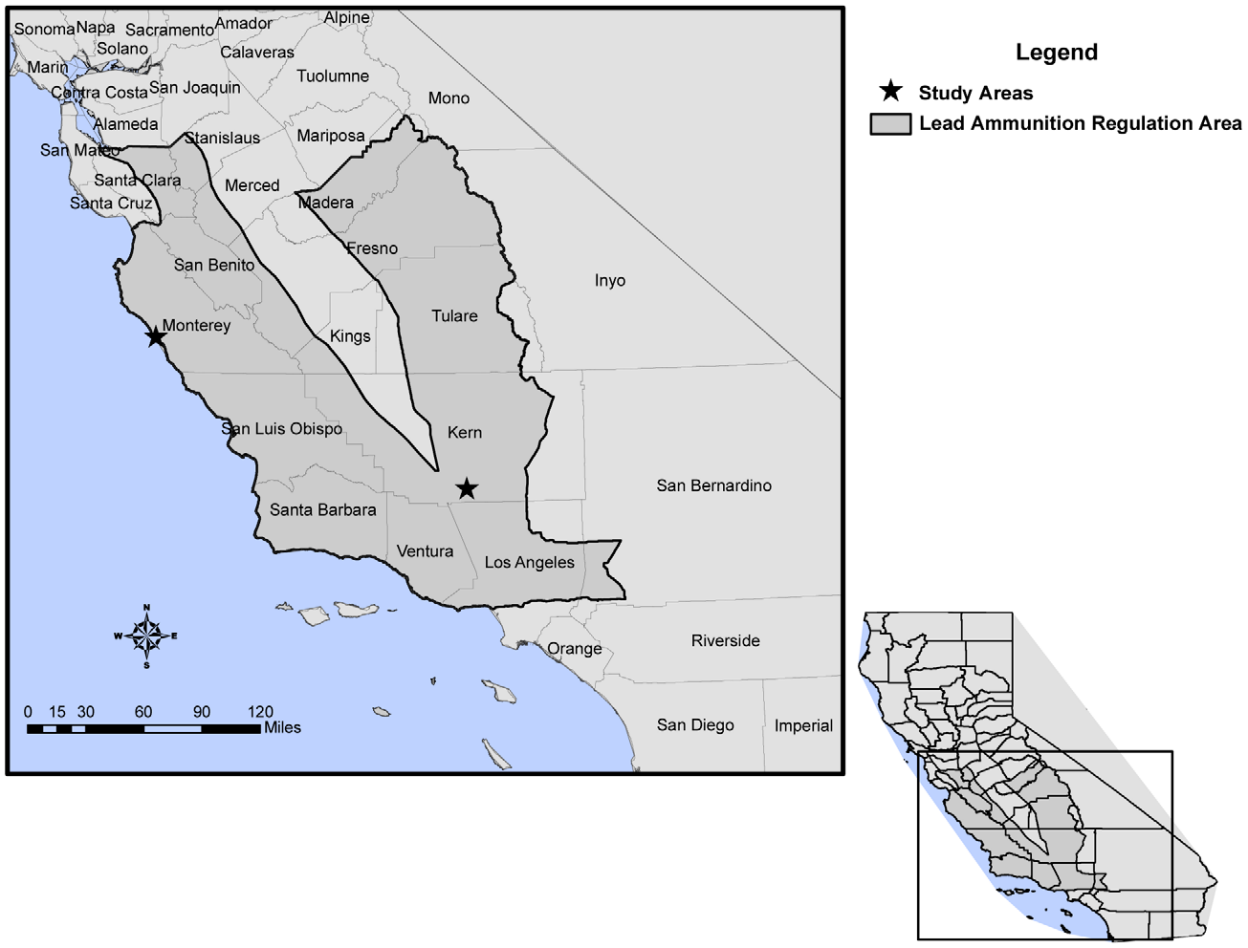
Location of study sites and area of lead ammunition regulation within the California condor range in California.

The California Fish and Game Commission adopted regulations on July 1, 2008 prohibiting the use of lead ammunition for hunting big game (deer, bear, wild pig, elk, and pronghorn antelope), and non-game species (coyote, ground squirrels, skunks, opossum, starlings, and other nongame wildlife) within the California range of the condor [24] (Figure 1). Restrictions against the use of lead ammunition for hunting upland game birds and small game mammals, such as rabbits and tree squirrels were not included in the ban.

We captured golden eagles on Tejon Ranch, a large tract of private land in Kern County (35°03′45″N, 118°41′18″W), which has one of the largest hunting programs in the state (Figure 1). During the 2007–2008 and 2008–2009 wild pig hunting seasons, Kern County was ranked second and first among counties in California for the numbers of wild pigs harvested, accounting for 26% and 18% of the total statewide harvest, respectively [25], [26]. Approximately 30% of the harvest in Kern County during the 2007–2008 season occurred on the private land where we trapped golden eagles [25]. Kern was also listed as one of the top two counties contributing to the statewide harvest of upland game birds (California quail and mourning doves), small mammal game (rabbits and tree squirrels), and nongame (coyotes, bobcats, and jackrabbits) animals in 2007. Deer and bear hunting in this area accounted for 2.7% and 3% of the total statewide harvests, respectively [27].

Eagles were sampled prior to the lead ammunition ban during the late fall/winter 2007 and late spring 2008, and following implementation of the ban in the late fall/winter 2008 and late spring 2009. Captures were performed during both seasons in order to assess lead exposure associated with different hunting activities, and account for seasonal differences in foraging and migratory behavior. The late fall/winter field seasons were concurrent with various hunting activities including big game (deer, wild pig, elk, and bear), small mammal game, upland game bird, and non-game (coyote, ground squirrels, and other nongame wildlife) hunting. The spring field seasons occurred during wild pig and non-game hunting. Eagles were observed to primarily forage on carrion during the late fall and winter sampling periods and spend more time hunting live prey during the spring. Late fall/winter captures were concurrent with golden eagle migration, so our sample likely included both local eagles and eagles recently arriving to the area from elsewhere. We weren't able to differentiate among eagles according to residency status during this time of year, so our late fall/winter samples were likely from a mixture of non-migrant and migrant eagles. This subset of eagles were classified as having unknown residency status. On the other hand, spring captures were not concurrent with golden eagle migration, so eagles sampled during this time period were classified as non-migrants. Additionally, eagles that were captured concurrently with the fall golden eagle migration, but re-sighted in our study area during the spring non-migratory season were re-classified as non-migrants.

We captured turkey vultures on the University of California Landels-Hill Big Creek Reserve in Monterey County (36°03′51″N, 121°34′28″W), an area surrounded by public and private land with high intensity wild pig hunting (Figure 1). Monterey County has the highest wild pig hunting pressure in California, accounting for greater than 18% of the total statewide reported pig harvest [27]. Vultures were sampled prior to the lead ammunition regulation in late spring 2008 and following implementation of the regulation in late spring 2009. Hunting activities, including wild pig and non-game (coyotes, ground squirrels, skunks, opossum, and starlings) hunting, were occurring at the time of captures and were within the daily flight range of turkey vultures from the study site. The seasons for upland game and small mammal game did not overlap with sampling activities at this site. Vultures were captured outside of the reported turkey vulture migration period [22] so that blood lead concentrations reflected local lead exposure.

### Sample collection and analysis

Turkey vultures were captured using a carrion baited walk-in trap with a live non-releasable “lure” vulture [28]. Carrion baited pit-traps [28] and bownets [29] were used to capture the golden eagles. The birds underwent basic health screening at the time of capture. Data collected on each individual included, sex, age class, body weight, and basic morphometric measurements. We categorized the age classes of vultures as hatch year (HY), second year (SY), and after second year (ASY) by coloration of the head and maxilla [30], and golden eagles as juvenile (1 year), subadult (2–5 years), and adult (>5 years) based on visible plumage characteristics [31]. Sex determination of the turkey vultures and golden eagles was performed using polymerase chain reaction analysis (Sex Made Easy™, Zoogen Incorporated, Davis, CA). Turkey vultures were marked using passive integrated transponders (AVID microchip system®, Avid Identification Systems, Inc., CA) to identify recaptured individuals. Golden eagles were banded with USGS metal rivet bands and marked with vinyl patagial tags in order to facilitate identification of individuals.

Blood samples for lead analyses were collected from the brachial vein within eight hours of capture into lithium heparin Microtainer blood tubes (Becton Dickinson, Franklin Lakes, NJ). The majority of blood samples were analyzed for lead concentration at the California Animal Health and Food Safety Laboratory (CAHFS), University of California, Davis using graphite atomic absorption spectrophotometry (PerkinElmer Model AAnalyst 800 graphite furnace atomic absorption spectrophotometer, PerkinElmer, Waltham, MA, USA). All samples were run in duplicate and results were considered acceptable when the relative standard deviation was ≤10%. The lower reporting limit for the lead in the blood samples for this laboratory was 6 µg/dL. A subset of the golden eagle samples were analyzed for lead concentration at the Environmental Toxicology Laboratory, University of California, Santa Cruz, for inclusion in a separate study, using inductively coupled plasma mass spectrometry (ICP-MS) (Finnigan MAT element magnetic sector-inductively coupled mass spectrometer, Thermo Fischer Scientific, West Palm Beach, FL, USA). All samples were run in duplicate and results were considered acceptable when the relative standard deviation was <1.5%. The lower reporting limit for the detection of lead in the blood samples analyzed at this laboratory was 1 µg/dL. We obtained additional golden eagle pre-regulation lead concentration data from a 1985–86 study assessing blood lead exposure in golden eagles captured in the same area as our Kern county eagle capture site [32]. Data were included if collected during the same time of year as our sampling (n = 91). Lead analyses for this study were performed using graphite atomic absorption spectrophotometry (PerkinElmer Model HGA 400 graphite furnace atomic absorption spectrophotometer, PerkinElmer, Norwalk, CT, USA). The lower reporting limit for detection of lead in the blood samples in this dataset was also 1 µg/dL [32].

A blood lead concentration of 10 µg/dL was used as a threshold value to differentiate “background” or “baseline” exposure (≤10 µg/dL) from elevated exposure (>10 µg/dL), which occurs with ingestion of lead from a point source. This threshold was chosen based on experimental lead dosing studies showing blood lead concentrations <10 µg/dL and <2 µg/dL in control bald eagles [33] and control turkey vultures [34], respectively, <4 µg/dL in captive California condors prior to release to the wild [35], and a median blood lead concentration of 1.8 µg/dL in free-flying common ravens sampled outside of the hunting season [36]. The depuration rate of lead (or half-time for lead elimination from blood) has been estimated to be approximately two weeks in condors [37] and less than two weeks in common ravens [36]. We therefore assumed that elevated blood lead concentrations in eagles and vultures captured post-ban during our study were reflective of lead exposure that occurred following implementation of the regulation.

### Data Analysis

We conducted independent analyses to assess the effect of the lead ammunition regulation on blood lead concentration in golden eagles and turkey vultures using the software package R [38]. Blood lead concentrations falling below the reporting limits (<1 µg/dL or <6 µg/dL) were reported by the laboratory as “nondetects” rather than numerical values, resulting in statistically “censored” data points. Probability plots and the Shapiro-Wilks test were used to assess the probability distribution of the blood lead concentration data. A significance level of 0.05 was used for all analyses unless specified otherwise.

### Golden eagles

The golden eagle data were analyzed using NADA (Nondetects And Data Analysis) [39], a library package in R that allows for censored data with multiple laboratory reporting limits to be incorporated into computations of statistics using nonparametric and parametric methods. Differences in blood lead concentration by sex and residency status (unknown or non-migrant) of golden eagles were evaluated using the Wilcoxon rank sum test. To assess the effect of age on blood lead concentration, age classes of golden eagles were compared using the Kruskal-Wallis test. In order to evaluate whether our smaller sample of pre-ban lead data was representative of lead concentrations in golden eagles prior to the ban on lead ammunition, blood lead concentration data derived from the 1985–86 study was compared to data from golden eagles sampled prior to the regulation for our study in 2007–2008 using the Wilcoxon rank sum test.

We used a censored linear regression model that assumes a lognormal distribution with maximum likelihood estimation to investigate the effectiveness of the lead ammunition ban on reducing blood lead concentration in golden eagles, while adjusting for important confounding variables, such as sex, age class, and residency status. For the model, age class was collapsed into two categories: subadult (juvenile and subadult age classes) and adult based on a lack of difference in blood lead concentrations between juvenile and subadult age classes in the univariate analyses. To identify the most parsimonious model, we used the likelihood-ratio test to determine whether each variable and interaction term significantly improved model fit (P≤0.1), compared to a model without that variable. Variables were retained in the model if they improved fit, while minimizing Akaike's information criterion (AIC), or were determined to be important confounders based on a 10% or greater change in the regression estimate for the lead ammunition ban variable with inclusion of the potential confounding variables in the model [40]. Overall model fit was assessed by evaluation of residual plots.

### Turkey vultures

Differences in blood lead concentration by sex and age class of the turkey vultures were evaluated as above for golden eagles using nonparametric statistical tests. We used a linear mixed effects model to investigate the effectiveness of the lead ammunition ban on reducing blood lead concentration in our sample of turkey vultures. Because fifteen turkey vultures were captured both before and after the ban, almost half of our sample consisted of repeated measures on individual vultures. These within-subject repeated measurements are likely to be correlated, so we used a linear mixed effects model to account for the non-independence in our data. Linear mixed effects models implement a likelihood based estimation method that allows for all available data to be used in the analysis while accounting for correlation and non-constant variability by including both fixed effect and random effect parameters. The NADA package does not offer an analytical framework for linear mixed effects models, so we used the nlme (Linear and Nonlinear Mixed Effects Models) library package in R [41] and substituted a value of one-half of the reporting limit for samples with lead concentrations falling below the reporting limit. Because the lead concentration data were not normally distributed, a logarithmic transformation was applied to the data. The data were then analyzed using a model that incorporated the presence of the lead ammunition ban as a fixed effect factor (binary), and subject identification (individual turkey vulture) as the random effect variable to account for the correlation in the repeated measurements. An unstructured covariance matrix was chosen for the random effect. The relative importance of adjusting for sex and age class as variables in the model was evaluated using the likelihood-ratio test to determine whether each significantly improved model fit (P≤0.1), compared to a model without that variable. Variables were retained in the model if they improved fit, while minimizing AIC, or were determined to be important confounders based on a change in the regression estimate for the lead ammunition ban variable by at least 10% with inclusion of the potential confounding variables in the model [40]. Overall model fit was assessed by evaluation of residual plots.

## Results

### Golden eagles

We captured a total of 55 golden eagles, with 17 eagles sampled prior to the ban and 38 eagles sampled post-ban. Fifteen eagles were captured or re-sighted in the same general area during the spring non-migratory season and were therefore classified as non-migrants. Golden eagle blood lead concentrations are summarized in Table 1. The prevalence of elevated lead exposure (>10 µg/dL) decreased 58%, from 76% (13/17, 95% CI: 53%–92%) pre-ban to 32% (12/38, 95% CI: 18%–48%) post-ban. In non-migrants, there was a 100% reduction in prevalence from 83% (5/6, 95% CI: 41%–99%) pre-ban to 0% (0/9, 95% CI: 0%–28%) post-ban. Blood lead concentrations in golden eagles sampled from 1985–86 were similar to concentrations in golden eagles sampled pre-ban for this study. Overall, there was no significant difference in lead levels between non-migrant golden eagles and eagles of unknown residency. However during the post-ban period, non-migrant golden eagles had significantly lower blood lead concentrations compared to eagles of unknown residency status (*P* = 0.04). While median blood lead concentration was not significantly different between subadult golden eagles (8 µg/dL) and adults (12 µg/dL) in our univariate analyses, age class was significantly associated with lead concentration in our multivariable analysis, and age class was included along with residency status and sex in the model to adjust for meaningful confounding and improve model fit (Table 2).

**Table 1:** Blood lead concentrations (µg/dL) in golden eagles sampled before and after the lead ammunition ban in southern California.

					Number of samples (%)				
Time period	Sample	Sample Size	Median (µg/dL)	Range (µg/dL)	>10 µg/dL	11–19 µg/dL	20–29 µg/dL	30–49 µg/dL	>50 µg/dL
Pre-ban (1985–1986)	All eagles	91	18	1–411	60 (66%)	18 (20%)	13 (14%)	23 (25%)	6 (7%)
Pre-ban (2007–2008)	All eagles	17	22	6–64	13 (77%)	4 (24%)	4 (24%)	4 (24%)	1 (6%)
	Non-migrants only	6	15	8–37	5 (83%)	3 (50%)	1 (17%)	1 (17%)	0
Post-ban (2008–2009)	All eagles	38	7	6–110	12 (32%)	5 (13%)	0	4 (11%)	3 (8%)
	Non-migrants only	9	6	6–10	0	0	0	0	0

**Table 2:** Regression estimates of the effect of the lead ammunition ban on blood lead concentrations (µg/dL) in golden eagles.

	Parameter estimate*	Standard error*	P-value
Intercept	3.84	0.42	<0.001
Lead ammunition ban (post-ban)	−1.01	0.31	0.001
Residency status (non-migrants)	−0.89	0.36	0.010
Age class (subadult)	−0.72	0.33	0.020
Sex (males)	−0.45	0.31	0.100

*Numbers presented on the natural logarithmic scale.

Based on our multivariable model, there was a significant reduction in golden eagle blood lead concentrations following implementation of the regulation, evidenced by a 3 fold decrease in concentrations from the pre-ban to post-ban period (*P* = 0.001, Table 2). Once the multivariate model accounted for the effect of all variables significantly related to lead concentration, we also detected a significant difference in blood lead concentrations between non-migrants and eagles of unknown residency, with levels in non-migrant golden eagles 2.5 times lower than concentrations in eagles of unknown residency status (*P* = 0.01).

### Turkey vultures

We captured a total of 71 vultures, with 38 turkey vultures sampled prior to the lead ammunition ban and 33 sampled post-ban. Fifteen of the vultures captured before the ban were recaptured the year following initiation of the regulation. Turkey vulture blood lead concentrations are summarized in Table 3. The prevalence of elevated blood lead exposure (>10 µg/dL) in the vultures decreased from 61% (23/38, 95% CI: 45%–75%) pre-ban to 9% (3/33, 95% CI: 2%–23%) post-ban, an 85% decline in prevalence. In recaptured individuals, the prevalence decreased 78%, from 60% (9/15, 95% CI: 35%–82%) pre-ban to 13% post-ban (2/15, 95% CI: 5%–45%). Blood lead concentrations did not differ by age or sex of the turkey vultures in our univariate analyses. Our linear mixed effects model demonstrated a significant decline (2.5 fold decrease) in blood lead concentration in turkey vultures following the lead ammunition ban (*P*<0.001, Table 4). Approximately one half of the unexplained variation originated from differences within individual vultures sampled both before and after the lead ammunition ban and the other half from differences between vultures. Despite a fairly large variation between turkey vultures, there was no significant difference in blood lead concentrations by sex and age class in our model.

**Table 3:** Blood lead concentrations (µg/dL) in turkey vultures sampled before and after the lead ammunition ban in central California.

					Number of samples (%)				
Sample	Time period	Sample Size	Median (µg/dL)	Range (µg/dL)	>10 µg/dL	11–19 µg/dL	20–29 µg/dL	30–49 µg/dL	>50 µg/dL
All vultures									
	Pre-ban (2008)	38	14	6–21	23 (61%)	16 (42%)	2 (5%)	5 (13%)	0
	Post-ban (2009)	33	6	6–44	3 (9%)	2 (6%)	1 (3%)	0	0
Recaptured vultures									
	Pre-ban (2008)	15	14	6–21	9 (60%)	4 (27%)	2 (13%)	3 (20%)	0
	Post-ban (2009)	15	6	6–44	2 (14%)	1 (7%)	1 (7%)	0	0

**Table 4:** Regression estimates of the effect of the lead ammunition ban on blood lead concentrations (µg/dL) in individual turkey vultures.

	Parameter estimate*	Standard error*	P-value
Intercept	2.44	0.1	<0.001
Lead ammunition ban (post-ban)	−0.99	0.14	<0.001

* Numbers presented on the natural logarithmic scale.

## Discussion

Blood lead concentrations significantly declined in both golden eagles and turkey vultures in the year following implementation of the lead ammunition ban, providing compelling evidence that the new regulation reduced lead exposure in these species. The analysis of lead exposure in turkey vultures, including repeated measures on individuals sampled both pre- and post-ban in our mixed model, documented a highly significant decline in blood lead concentration post-ban. These findings indicate that there has been a positive impact of the lead ammunition ban on reducing lead exposure in individual vultures sampled for our study. Analyses of golden eagle data also demonstrated a significant reduction in lead exposure after the ban on lead ammunition, which indicates that the lead ammunition ban can be effective in decreasing lead exposure across multiple scavenging bird species. The reduction in lead exposure was much greater for our subset of non-migrant eagles compared to the overall sample which most likely included eagles originating from outside of the banned area that may have ingested lead contaminated carcasses prior to migrating into our study area.

Our analyses of golden eagle lead exposure also showed that blood lead concentration was significantly higher in adults compared to subadults. There are a number of studies demonstrating higher blood and tissue lead concentrations in older birds [42]–[46] suggesting that age-related differences in blood and tissue lead concentration may be the result of dissimilarities in lead uptake into bone with enhanced uptake in growing birds with ossifying bone [46], [47] or accumulation of body burdens of lead with increasing age [45], [46]. We did not observe differences in foraging behavior between age classes of golden eagles that would contribute to this variation.

According to available hunter-tag return data for the 2007–2009 period, statewide wild pig and deer hunting pressure was fairly constant across the pre- and post-ban periods. The number of deer harvested on Tejon Ranch increased in the 2008 post-ban season compared to the 2007 pre-ban season [48], [49], while the county level data for pig harvests showed a decrease in the number of wild pigs harvested in Kern and Monterey counties in 2008–2009 compared to 2007–2008 [26], [27]. A decrease in wild pig hunting pressure during our study period may have contributed to a decrease in post-ban lead exposure in golden eagles and turkey vultures, but hunter-return pig tag data indicate that there was still substantial pig hunting occurring in Kern (705 tags returned) and Monterey (640 tags returned) counties in 2008–2009 after the ban was implemented [26], [27]. The very low lead exposure we observed in turkey vultures and golden eagles captured post-ban, despite relatively high hunting pressure in these two counties, suggest there was extensive hunter compliance with the ban on lead ammunition in these study areas.

Reduced but persistent post-ban lead exposure in eagles was most commonly detected in eagles of unknown residency status, which may have been due to lead exposure occurring in areas not covered by the ban. Ongoing lead exposure incidents in eagles and turkey vultures may also be explained by the use of lead ammunition for hunting activities not included in the regulation or less than full compliance. Harvest of upland game birds and small game mammals, which was not included in the ban on lead ammunition, was occurring during our late fall/winter golden eagle captures. Unlike non-game animals that are hunted and usually left in the field, game species must be retrieved by law, and are therefore assumed to be less likely to act as a source of lead exposure to condors and other avian scavengers. However, a fraction of these animals are wounded and not retrieved, and as a result may be a source of lead to scavengers [50]. We expected that compliance with the lead ammunition ban would have been limited in the first year following implementation, especially because non-lead ammunition for hunting small mammals, such as ground squirrels and jackrabbits, was not readily available when the ban was first implemented. Predatory and scavenging birds often feed in pairs or flocks, so even a few lead contaminated carcasses or animal remains can provide a source of lead exposure for a substantial number of individuals.

Clinical signs associated with lead toxicity were not observed in any of our birds, although this may be difficult to assess in the field setting. In this study, 53% of golden eagle and 18% of turkey vultures sampled prior to implementation of the ban, and 18% of the golden eagles and 3% of turkey vultures sampled post-ban had blood lead concentrations consistent with subclinical lead toxicity (>20 µg/dL) [51]. Only one captured golden eagle had a blood lead concentration at a level that has been reported to cause lead poisoning and death in raptors (>100 µg/dL) [51]. Sampling of free-ranging birds using the capture methods we employed here may underestimate burdens of lead exposure and poisoning in scavenging and predatory bird populations, especially for birds with blood lead concentrations that are high enough to cause debilitation and preclude birds from flying and searching for food [52].

Our southern California study site where we sampled golden eagles was located in an area with an intensively managed hunting program, which may have contributed to the 100% reduction in elevated lead exposure in non-migrant eagles at this site. This finding may not be representative of other hunting seasons or other areas in California where hunting is not as heavily monitored. Similar actions have been taken elsewhere to regulate or encourage hunters to utilize non-lead ammunition for hunting in order to protect susceptible scavenging bird populations. In response to lead poisoning of white-tailed eagles (*Haliaeetus albicilla*) resulting from feeding on game hunted with lead bullets in northeastern Germany [53], [54], lead ammunition was prohibited for game hunting in federal forests. Additionally, an interdisciplinary research program involving local stakeholders was established to expand use of non-lead ammunition in areas outside of national forests where this population is affected and generate other feasible solutions to this problem, including burying lead-contaminated discarded viscera [55]. Measures have also been taken on the island of Hokkaido, Japan where significant mortality in white-tailed eagles and Stellar's sea-eagles (*Haliaeetus pelagicus*) has been attributed to feeding on hunter killed sika deer (*Cervus nippon*) [56]. Local authorities have banned the use of lead ammunition for hunting on the island since 2001 in response to this problem [56], but to our knowledge, information regarding the effectiveness of these efforts has not yet been published. Additionally, the Arizona Game and Fish Department has promoted the voluntary use of non-lead ammunition for hunting within the condor range in Arizona since 2003. These efforts led to a decrease in condor lead exposure and a reported 80% hunter compliance during the 2007 hunting season [57].

Our findings provide direct evidence that regulating the use of lead ammunition for hunting can reduce lead exposure in predatory and scavenging birds. Since the initiation of the ban in 2008, ammunition manufacturers have increased their production of non-lead ammunition in response to demand, and numerous non-lead ammunition alternatives are now available for hunting both small and large game and non-game species [58]. Replacement of lead ammunition with non-lead alternatives will greatly reduce the risk of lead poisoning and associated mortality in predatory and scavenging birds, and may benefit the conservation of these species.
